# Hantavirus Infections among Military Forces

**DOI:** 10.1093/milmed/usad261

**Published:** 2023-07-10

**Authors:** Jukka Mustonen, Heikki Henttonen, Antti Vaheri

**Affiliations:** Faculty of Medicine and Health Technology, Tampere University, Tampere 33014, Finland; Department of Internal Medicine, Tampere University Hospital, Tampere 33520, Finland; Wildlife Ecology, Natural Resources Institute Finland, Helsinki 00790, Finland; Department of Virology, Medicum,, University of Helsinki, Helsinki 00290, Finland

## Abstract

**Introduction:**

Hantaviruses cause two kinds of clinical syndromes. Hemorrhagic fever with renal syndrome is caused by Hantaan virus in Asia, Puumala virus (PUUV) and Dobrava virus in Europe, and Seoul virus worldwide. Hantavirus cardiopulmonary syndrome is caused by Sin Nombre virus in North America and Andes virus and related viruses in Latin America. All hantaviruses are carried by rodents and insectivores. Humans are infected via inhaled aerosols of rodent excreta. In the history, there are several epidemics of acute infectious diseases during many wars, which have been suggested or proven to be caused by various hantaviruses.

**Materials and Methods:**

Literature review of 41 original publications and reviews published between 1943 and 2022 was performed. Among them, 23 publications handle hantavirus infections among military forces, and the rest 17 hantavirus infections themselves.

**Results:**

A large epidemic during World War II in 1942 among German and Finnish soldiers in Northern Finland with more than 1,000 patients was most probably caused by PUUV. During Korean War in 1951–1954,∼ 3,200 cases occurred among United Nations soldiers in an epidemic caused by Hantaan virus. During Balkan war from 1991 to 1995, numerous soldiers got ill because of hantavirus infection caused by PUUV and Dobrava virus. Several other reports of cases of various hantavirus infections especially among U.S. soldiers acting in South Korea, Germany, Bosnia, and Kosovo have been described in the literature.

**Conclusions:**

Military maneuvers usually include soil removal, spreading, digging with accompanied dust, and living in field and other harsh conditions, which easily expose soldiers to rodents and their excreta. Therefore, the risks of hantavirus infections in military context are obvious. All military infections have been caused by hantaviruses leading to hemorrhagic fever with renal syndrome.

## HISTORICAL BACKGROUND

As reviewed by Heyman and associates,^1^ clinical syndromes possibly caused by hantaviruses were noted in China dating back to the first millennium and hantavirus disease has been suggested as a possible cause for the 1862–1863 “trench nephritis” epidemic during the American Civil War. It was also mentioned that in Korea, Manchuria, and Far-Eastern Russia, the problem was probably endemic for centuries, although it was first mentioned in the Vladivostok region hospital in 1913–1914.^1^

It has been suggested that trench nephritis during World War I in 1914–1918 was caused by hantavirus.^1,2^ The disease was clinically characterized by breathlessness, swelling of the face or legs, or generalized dropsy.^3,4^ Most patients were afebrile and hypertensive.^4^ The clinical picture, however, was totally different compared with that observed in patients with hantavirus infections.^5^ In postmortem studies, the kidneys were typically swollen, but in contrast to findings in hantavirus infections, kidneys were pale rather than hemorrhagic. The renal histopathologic findings showed an acute glomerulonephritis.^4^ The morphologic renal lesion in hantavirus infections is acute hemorrhagic tubulointerstitial nephritis with only mild glomerular changes.^6^ Based on the available evidence, it appears that trench nephritis was most probably not caused by hantavirus.

A new disease was observed among the Japanese and Russian armies at the boundary between Manchuria and Soviet Union in 1932.^7^ Extensive clinical and epidemiological studies were performed, and the disease was concluded to be a viral infection. Both Russian and Japanese workers succeeded in producing the disease in human volunteers by intravenous injection of blood or urine from the patients, but they could not establish the disease in experimental animals.^7^ During the past decades, the Russians and Japanese have used various names for the disease including epidemic hemorrhagic nephrosonephritis (Russia) and Manchurian hemorrhagic fever or Songo fever (Japan), but nowadays, the term hemorrhagic fever with renal syndrome (HFRS) is usually used. We do not surely know whether these diseases were caused by hantaviruses.

## PUUMALA HANTAVIRUS EPIDEMIC DURING WORLD WAR II IN NORTHERN FINLAND

A large epidemic of acute infectious disease occurred in the front of Salla, Eastern Lapland, Finland, in 1942. About 1000 German and 60 Finnish soldiers got ill. It is not known whether there were cases also among the soldiers of Soviet Union. A German medical doctor Kondrad Stuhlfauth reported about the epidemic in German language in 1943,^8^ and a Finnish doctor Herman Hortling published a report in Finnish in 1944^9^ and in Swedish in 1946.^10^

Both Stuhlfauth^8^ and Hortling^9,10^ regarded the disease as previously unknown. Most probably, they were not aware of two publications from Sweden in 1934. Myhrman^11^ described seven hospital-treated patients in Central Sweden, and Zetterholm^12^ reported also of seven patients from Northern Sweden. Later in 1945, Myhrman suggested the name “nephropathia epidemica” to a new disease.^13^ In 1980, Brummer-Korvenkontio, Vaheri, and associates^14^ reported that the disease was caused by a virus transmitted by a bank vole (*Myodes glareolus*) and the virus was named Puumala virus (PUUV).

According to Stuhlfauth,^8^ leptospirosis was suspected, but it could not be proven. The primary reports concluded that the disease did not spread through human contact. Among orthohantaviruses, person-to-person transmission has been documented only in infections caused by Andes virus in South America.^5^

Many other clinical findings of the soldiers were likewise quite typical for PUUV infection. They included high fever, headache, abdominal and back pains, nausea, and vomiting. Some patients had hypotension.^8,9^ All these symptoms have since been found in serologically confirmed patients with PUUV infection in Finland.^15^

Of particular interest were the ocular symptoms. Transiently reduced vision was reported in 25% of the patients.^8^ This symptom described by Stuhlfauth as “accommodation cramp” was also a new finding to the doctors of the armies. It is quite specific to hantavirus infections, and its prevalence in later studies has varied from 12 to 36% of the patients.^15–17^ The laboratory findings were transient proteinuria, hematuria, and reduced renal function, with all these being typical for acute kidney injury in PUUV infection.^6^

The clinical course of the patients was favorable. Almost all soldiers were able to come back to their units, and no fatal cases occurred. The case fatality rate in PUUV infection is very low, and the reported frequencies range from <0.1% in Finland to 0.4% in Sweden.^5^

Stuhlfauth^8^ pays attention to the fact that 74% of the sick soldiers were in the front lines, obviously in poor living conditions. The proportion of sick soldiers declined behind the front lines. Both Stuhlfauth^8^ and Hortling^9,10^ specifically highlight the abundance of rodents. Hortling: “Summer 1942 was characterized by unusual abundance of mice and lemmings. Already in spring 1942, these rodents were commonly met in trenches and ground cabins all around.” In the course of the 4-year rodent cycle in Lapland, lemmings and other rodents increased in 1941 and 1942 was the cyclic peak year in abundance.^18^

The contemporary articles refer to mice. Even if there are several rodent species in Lapland (voles and lemmings), there are no real mice, like yellow-necked mouse (*Apodemus flavicollis*) or wood mouse (*Apodemus sylvaticus*) in Lapland. The only rodent species known to carry a hantavirus in Finland is the bank vole hosting the PUUV. Bank vole is commonly found in the whole of Finland except for the northernmost mountainous regions above the conifer forest line. The epidemic region Salla therefore belongs to the permanent range of the bank vole. PUUV is common in the bank vole up to the northern limit of the host.^19,20^

The year 1942 was also a peak year of Norway lemmings (*Lemmus lemmus*) in eastern Lapland, and this has raised speculations if the lemmings could have been the source of the epidemic. We have isolated Topografov orthohantavirus from Siberian lemmings (*Lemmus sibiricus*) in Taimyr Peninsula in Siberia. However, we have never found any hantavirus in Norway lemmings in Fennoscandia.^21,22^ Furthermore, Topografov orthohantavirus is not known as a zoonotic human pathogen. We have also tried in various ways to infect lemmings with PUUV but with no success. It thus seems unlikely that PUUV from bank voles could have jumped to lemmings and cause an epidemic through a host switch.

Some Finnish veterans from Salla front have been studied. Serum samples collected 50 years later contained hantaviral antibodies, but, because of the long interval, the specificity of these antibodies could not be defined anymore, i.e., it was not possible to conclude whether the cause was PUUV or some other hantavirus. Neither do we have detailed clinical information of the veterans during the war time nor afterward.

All available facts of the Salla epidemic among German and Finnish soldiers in 1942 in Eastern Lapland confirm that it was caused by PUUV, spread by bank voles^23^. Consequently, the reports written by Dr Stuhlfauth in 1943 in Germany^8^ and by Dr Hortling in 1944 in Finland^9,10^ were the first publications on PUUV infection in these countries, although the rodent host species were not known at that time. This is the largest local epidemic of PUUV infection ever described in the literature.

## HANTAAN VIRUS EPIDEMIC IN KOREAN WAR

HFRS came first to the attention of western physicians when ∼3200 cases occurred from 1951 to 1954 among United Nations soldiers in Korean War.^24,25^ Such an incidence of a serious disease constituted an important military problem.^26^ Not only most cases occurred as isolated events, but also small outbreaks emerged in the troops. The disease seemed to be noncontagious. The clinical picture included sudden intense headache, fever, and chills as well as anorexia and vomiting. Petechial rash and episodes of hypotension were present in some patients. Leukocytosis, thrombocytopenia, albuminuria, and elevated serum creatinine level were typical laboratory findings. The case fatality rate was at first well over 10%, but it dropped to about 5%.^9^ Hemorrhages in the kidneys, the pituitary, the adrenal, and the right auricle of the heart were found at autopsies.^26^

The American Army medical service established a Hemorrhagic Fever Center close to the region in which the largest number of cases occurred in South Korea.^7^ All suspected cases of the disease were evacuated by a helicopter to the Center’s hospital where a careful investigative program and the best treatment of the patients were conducted.

Despite much research, the agent of the disease, Korean hemorrhagic fever (KHF), remained unknown until 1978, when a new virus, Hantaan virus (HTNV), named after the Hantaan river, was isolated in its rodent host, striped field mouse (*Apodemus agrarius*).^27^ A retrospective analysis of sera collected from soldiers during the Korean conflict confirmed that KHF was caused by HTNV.^28^

According to Gajdusek,^7^ there have been several deaths from the disease in the United States in military personnel who returned from Korea to America during the long incubation period of 2-6 weeks in HFRS. In a follow-up study, Korean War veterans were followed until December 31, 1998. The results showed that KHF did not increase mortality rates, but two outcomes, transient ischemic attacks and diabetes, were significantly associated with increased morbidity rates for non-Caucasian cases.^29^

## PUUMALA AND DOBRAVA VIRUS INFECTIONS IN BALKAN WAR FROM 1991 TO 1995

During the 1987–2001 period, 235 cases of HFRS were recorded in Croatia and 147 (63%) of them were among Croatian army soldiers.^30^ The causative agents included PUUV and Dobrava virus (DOBV). During the epidemic in 1995, there were 129 HFRS cases, of which 120 were soldiers. The soldiers were typically accommodated in wooden huts in beech forests, a typical habitant for abundant local hantavirus carrier rodents, bank vole (PUUV), and yellow-necked mouse (DOBV).^30^

The war in Yugoslavia caused massive streams of refugees out of Kosovo region, which ended up in refugee camps in Macedonia (North Macedonia), Albania, and Montenegro.^31^ Obviously, also, those people in the camps were exposed at risk to acquire hantavirus infections.^31^

## OTHER REPORTS OF HANTAVIRUS INFECTIONS AMONG MILITARY FORCES

There are many reports from several countries about HFRS cases among military personnel not in actual war situations.^32^ Fourteen of 3754 U.S. mariners who participated in a joint United States–Republic of Korea training exercise in 1986 developed HFRS (33). Ten soldiers were hospitalized, and two of them died. No subclinical infections were identified. The outbreak is the largest cluster of HFRS cases among U.S. personnel in the Republic of Korea since the Korean conflict.^33^

According to Song and associates,^34^ four U.S. soldiers acquired HFRS caused by HTNV while training near the demilitarized zone, South Korea, in 2005. The total number of soldiers was not mentioned in the report. The genome of HTNV sequences obtained from patients was identical to a viral sequence from striped field mice captured in the same area.

In Sweden, a total of 705 soldiers involved in field training in three PUUV-endemic counties were bled twice within a 6-month interval.^35^ Three soldiers seroconverted when tested for PUUV antibodies. Mild febrile episodes were recorded in two of them. It was concluded that military populations are at considerably greater risk of getting PUUV infection when compared to the entire population in the same area.^35^

Hantavirus outbreak during military maneuvers among U.S. troops occurred in Germany in 1990.^36^ A total of 16 cases of HFRS caused by PUUV were found. Risk factors for the disease were “sighting of rodents” and “using hay in sleeping areas.”^36^

Hukic and associates^37^ reported in 1996 that during the past 12 months, more than 300 patients with HFRS have been admitted to Tuzla hospital in North-East Bosnia. Several factors such as the presence of military camps with large amounts of food stored under primitive conditions, inadequate garbage disposal, or the general breakdown of hygiene caused by water and power shortage may have resulted in a higher density of rodents. The authors confirmed two cases of HFRS among the multinational United Nations forces.^37^

A case report described HFRS caused by DOBV in an active duty U.S. soldier in Kosovo in spring 2013.^38^ The patient had typical clinical symptoms and laboratory findings of HFRS. There were no other soldiers in his unit with similar symptoms.

## UKRAINE WAR

Concerning the war in Ukraine, it has recently been reported that pathogenic hantaviruses are present in some common rodent species in Ukraine.^39^ The incidence of HFRS cases in humans in Ukraine remains unknown, but 1.6% of healthy individuals in the country have antibodies to hantaviruses.^40^ It is quite possible that hantavirus infections will emerge among the military troops and civilians during the ongoing war in Ukraine.

## IMPORTANCE OF HANTAVIRUS INFECTIONS

The conditions in war frontiers include several risk factors for hantavirus infections. Prevention of exposition to rodents and their excreta reduces the risk of infections. Such efforts include the use of traps and poisons, elimination of rodent food sources, measures to prevent rodents entering into houses, ventilations of rooms, and use of rubber gloves, disinfectants, and masks. Rodent fluctuations can be monitored, and high-density risk periods have been predicted. At the demilitarized zone at the borders of South Korea and North Korea, rodent and hantavirus situation is regularly monitored specifically from the military point of view.^41^ To our knowledge, there are no preventive measures that have been shown to be effective among soldiers.

At present, there are no hantavirus vaccines acceptable by western standards, and there is no specific therapy for hantavirus infections. It seems to us that hantavirus infections have not played a main role in the outcomes of military operations. It, however, is quite important that military doctors know the main clinical symptoms of these infections and perform reliable serologic diagnostics. Unnecessary therapeutic procedures including antibiotic therapies can be avoided, and patients who need hospital treatment can be identified.

## CONCLUSIONS

Large epidemics of hantavirus infections occurred among German and Finnish soldiers in Finnish Lapland in 1942 and among United Nations soldiers during Korean War from 1951 to 1954. A small number of patients with hantavirus disease have been documented during Balkan war from 1991 to 1995. In addition, there are many reports from several countries about HFRS cases among military personnel not in actual war situations.

## Data Availability

The data that support the findings of this study are available on request from the corresponding author. All data are freely accessible.

## INSTITUTIONAL CLEARANCE

Does not apply.
